# Preference for the Nearer of Otherwise Equivalent Navigational Goals Quantifies Behavioral Motivation and Natural Selection

**DOI:** 10.1371/journal.pone.0054725

**Published:** 2013-01-23

**Authors:** Russell E. Jackson

**Affiliations:** Psychology and Communications Studies Department, University of Idaho, Moscow, Idaho, United States of America; Institut Pluridisciplinaire Hubert Curien, France

## Abstract

Navigation and environmental perception precede most actions in mobile organisms. Navigation is based upon the fundamental assumption of a ubiquitous Preference for the Nearest of otherwise equivalent navigational goals (*PfN*). However, the magnitude and triggers for *PfN* are unknown and there is no clear evidence that *PfN* exists. I tested for *PfN* in human participants on a retrieval task. Results of these experiments provide the first evidence for *PfN*. Further, these data quantify the three primary *PfN* triggers and provide an experimental structure for using *PfN* as a behavioral metric across domains. Surprisingly, *PfN* exists at a high, but not universal, magnitude. Further, *PfN* derives most from the absolute distance to the farthest of multiple goals (*d_f_*), with little influence of the distance to the nearest goal (*d_n_*). These data provide previously unavailable quantification of behavioral motivation across species and may provide a measurable index of selection. These methods hold particular import for behavioral modification because proximity is a powerful determinant of decision outcomes across most behaviors.

## Introduction

Environmental perception forms the basis for essential behaviors including foraging, dispersal, territoriality, and mating [1]–[3]. Environmental perception, and distance perception in particular, comprises the foundation of navigation in natural and artificial systems [4]–[7]. Environmental perception and navigation precede most human and nonhuman animal activity and so their expression holds the potential to affect most behaviors.

Researchers assume that navigation is based on a Preference for the Nearest of otherwise equivalent navigational goals (*PfN*). Navigation and perception researchers often assume the existence of a *PfN* as a means of avoiding costly navigation [8]–[10]. Further, entire fields of study across disciplines as diverse as geography, neuroscience, and cinema are based upon an assumption that nearness indicates likelihood of inclusion. Proxemics, the “mere-exposure effect” [11], vision science (e.g., ideal observer analysis [12]), cognitive or mental mapping [13], and artistic depictions of desirability all use *PfN* as a foundational construct. Across the behavioral sciences, the assumed *PfN* would directly affect most locomotor behavior across species, and indirectly affect any behavior during which navigation or environmental perception occur.

Unfortunately, no research clearly demonstrates a navigational *PfN*. This lack of empirical data undermines conclusions from the considerable research based upon assumed *PfN*. Some evidence suggests that organisms do not universally navigate to the nearest navigational goal [14]. Even if a *PfN* exists, we do not know its magnitude. Research based on the assumption of *PfN* often assumes a universal magnitude–that humans and other species always prefer nearer goals, even if only trivially nearer [15]. If *PfN* exists, we also do not know what triggers initiate it. In situations with multiple navigational options, *PfN* could be triggered by the distance to the nearest goal (*d_n_*), the farthest goal (*d_f_*), or the difference in distance between goals (*d_f−n_*); however, no previous research has even acknowledged that there are at least three such dimensions. Inability to determine or quantify *PfN* currently prohibits quantitative empiricism in this fundamental area.

Human participants in the current study completed a navigational retrieval task in two psychophysical experiments designed to detect and quantify *PfN*. I designed this procedure in order to provide a method for quantifying navigational preference in a way available across species. The goal of this analysis was to determine if *PfN* existed and if a mathematical function could capture its variability across changes in *d_n_*, *d_f_*, and *d_f−n_*.

## Results

*PfN* existed at a high, but surprisingly not universal, magnitude. Magnitude *PfN* equals the proportion of trials in which the participant selected the nearer goal. Participants exhibited *PfN* in 79% (1258/1590) of trials, excluding trials with equidistant goals. Thirty percent (32/106) of participants exhibited *PfN* in all trials.

**Figure 1:**
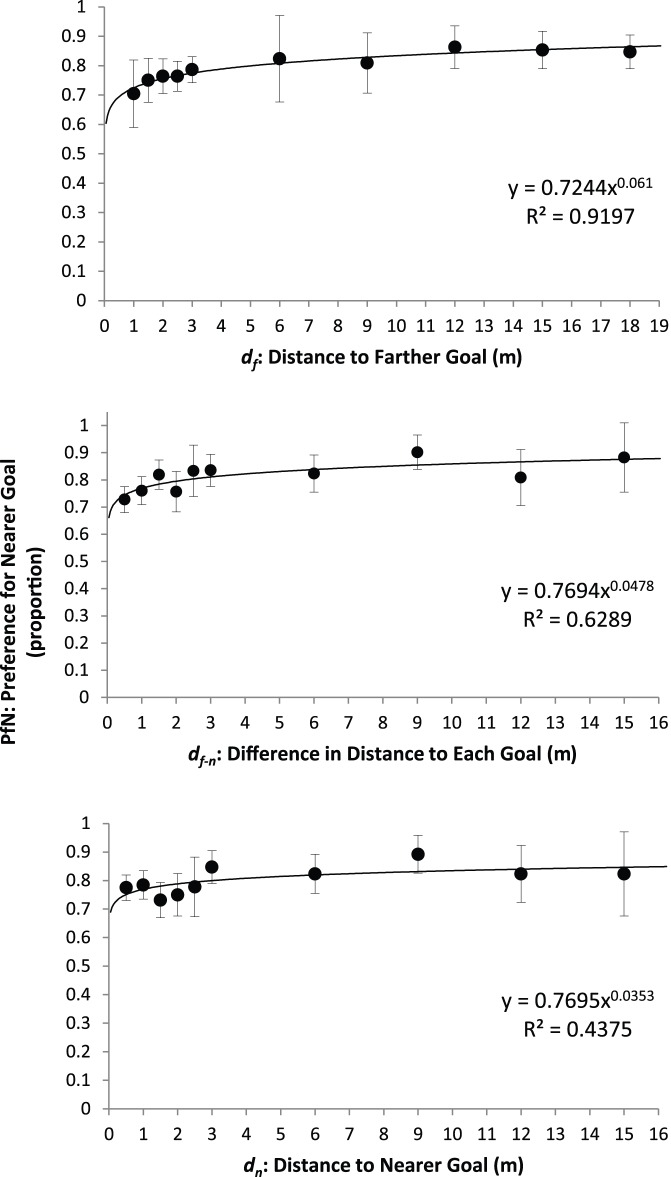
Proportion of *PfN* predicted by *d_f_*, *d_n_*, and *d_f−n_*. Error bars represent 95% confidence intervals about the mean.

Contrary to intuitive expectations, distance to the nearer goal (*d_n_*) affected navigational choice weakly, but distance to the farther goal (*d_f_*) predicted *PfN* strongly, with only moderate impact of distance difference between goals (*d_f−n_*). Figure 1 graphs the average participant *PfN* across distances for each of the three predictors. The primary goal of this analysis was to identify a function that would best account for variance in those averages for each of the three predictors. Considered independently, an exponential function of *d_f_*, *PfN* = 0.724 *d_f_* ^0.061^, explained a very large amount (*R^2^* = 0.920) of the variance in average participant *PfN* across distances. An exponential function of *d_f-n_*, *PfN* = 0.769 *d_f-n_* ^0.048^, explained a moderate amount (*R^2^* = 0.629) of *PfN* variance, while *d_n_*, *PfN* = 0.770 *d_n_* ^0.035^, explained a small amount (*R^2^* = 0.438) of *PfN* variance.

## Discussion

These data provide the first substantive evidence for, and quantification of, *PfN*. The function of all three *PfN* triggers was asymptotic, allowing normal (Gaussian) approximations over any distance. This allows quantification of *PfN* as a parameter under Stephen’s Power Law (or Weber’s Law) and opens this area to applications of Signal Detection Theory [16].

An important contribution of this study is that the featured research design can determine the relative navigational impact of additional variables, such as determining how the functions of each trigger change with fatigue or determining the relative impact of hunger when navigational goals include food. The resource that *PfN* conserves could include time, energy, exposure to risk, or other factors that likely vary across scenario and it would be important to weigh these factors against a navigational preference, versus the perceptual capacity to detect distance differences. Similar decision rules may inform artificial systems for automated robotic navigation. A valuable direction in this research will be to investigate covariance between additional variables and between *d_f_*, *d_n_*, and *d_f−n_* in the particular settings in which researchers test additional variables. Variance in the mathematical function of the three *PfN* triggers across scenarios provides a method of quantification unlimited by discipline or technique.

This work provides a method for quantifying behaviors and motivations. The distance that an organism is willing to navigate in order to attain a goal provides a metric for measuring the relative worth to that organism. For example, the extra distance that a sparrow flies in order to nest at a better site gives a precise measurement of the difference in worth between sites to the sparrow (calculable from the function of *PfN* from *d_f_*, *d_f−n_*, and *d_n_*). This is an important contribution toward the difficult quantification of motivations in animals.

Applications of this approach exceed animal behavior and may provide a means of quantifying selection pressure. For example, when considering the costs of buoyancy, the distance that buoyant seeds travel can approximate the reproductive benefit (selective advantage) of broad dispersal in that plant (i.e. negative *PfN*), wherein the strength of (negative) *PfN* would indicate the strength of selection. Specifically, studying reproductive output as a product of spatial dispersion (with known dispersal costs) quantifies selection pressure in any situation where reproductive output varies spatially across organisms. Given the broad applications for investigating spatial dispersal of organisms, calculating *PfN* may provide a broad method of quantifying selection. These data provide a baseline for calculating the relative worth of strategies across species and enable quantification in areas previously unavailable to empirical investigation, including potential quantification of the response to natural selection.

Data in the current scenario suggested that *PfN* may be better conceptualized as an avoidance of the farthest navigational goal, rather than preference for the nearest. The extent to which this generalizes to other areas could hold important implications across disciplines. For example, some ideal-observer analyses may function more through the of exclusion of distant targets, rather than the inclusion of near ones. The “mere exposure effect” may be better understood as xenophobic avoidance of unexposed targets, rather than as an attraction toward common targets. Distal, rather than proximal, locations in mental or cognitive maps may best determine the strength of relationships between elements.

*PfN* is not absolute, but very high. *PfN* determined the majority of navigational choice between functionally identical navigational goals. The outcomes of many human decisions as diverse as selecting a doctor or one’s next meal may be byproducts of navigational proximity. Further, the capacity to alter such behaviors might benefit most from altering the navigational scenario. For example, the most effective method of increasing mass transportation usage might focus on advertising the time savings over personal vehicle usage. Altering apparent travel is a promising cue for behavioral modification because proximity is a powerful determinant of decision outcomes.

## Methods

### Ethics Statement

All participants gave written informed consent prior to participation and the Institutional Review Board at California State University San Marcos approved all methods prior to data acquisition.

Each participant stood in a fixed starting location near the middle of a roughly 61 m hallway at the beginning of each trial. A research assistant placed two 7 cm diameter spheres on the ground at a distance (described below) on either side of the participant. Participants selected one sphere, retrieved it, and then returned to the starting position. Seventy-two participants in Experiment 1 retrieved spheres placed at 0.5 m increments up to 3.0 m away from the participant, while thirty-four participants in Experiment 2 retrieved spheres placed at 3.0 m increments up to 18 m away. Research assistants placed the spheres at every possible unique permutation of distances (21 trials/participant). For example, for every given distance to one sphere (e.g. 0.5, 1.0, 1.5, 2.0, 2.5, or 3.0 m in Experiment 1), participants received a trial with every possible distance to the other sphere. Research assistants presented a randomized order of trials, but without the same distance in consecutive trials, and also followed a randomization structure for the side on which any given distance appeared (left or right). Participants also completed a short demographics questionnaire.
